# Structural Changes of Lignin after Liquid Hot Water Pretreatment and Its Effect on the Enzymatic Hydrolysis

**DOI:** 10.1155/2016/8568604

**Published:** 2016-08-03

**Authors:** Wen Wang, Xinshu Zhuang, Zhenhong Yuan, Wei Qi, Qiang Yu, Qiong Wang

**Affiliations:** ^1^Key Laboratory of Renewable Energy and Guangdong Key Laboratory of New and Renewable Energy Research and Development, Guangzhou Institute of Energy Conversion, Chinese Academy of Sciences, Guangzhou 510640, China; ^2^Collaborative Innovation Center of Biomass Energy, Zhengzhou 450002, China

## Abstract

During liquid hot water (LHW) pretreatment, lignin is mostly retained in the pretreated biomass, and the changes in the chemical and structural characteristics of lignin should probably refer to re-/depolymerization, solubilization, or glass transition. The residual lignin could influence the effective enzymatic hydrolysis of cellulose. The pure lignin was used to evaluate the effect of LHW process on its structural and chemical features. The surface morphology of LHW-treated lignin observed with the scanning electron microscopy (SEM) was more porous and irregular than that of untreated lignin. Compared to the untreated lignin, the surface area, total pore volume, and average pore size of LHW-treated lignin tested with the Brunner-Emmet-Teller (BET) measurement were increased. FTIR analysis showed that the chemical structure of lignin was broken down in the LHW process. Additionally, the impact of untreated and treated lignin on the enzymatic hydrolysis of cellulose was also explored. The LHW-treated lignin had little impact on the cellulase adsorption and enzyme activities and somehow could improve the enzymatic hydrolysis of cellulose.

## 1. Introduction

Bioethanol production from lignocellulosic biomass has gained focus for easy availability of feedstock, no competition with the food supply, and reduction in net carbon emission [1, 2]. Cellulose, hemicellulose, and lignin which are the main components of lignocellulosic materials form complex and compact structure via covalent and noncovalent bonds to make lignocellulose resist the microbial and enzymatic attack [1]. Pretreatment followed by enzymatic hydrolysis and fermentation is an essential process for the effective bioconversion of lignocellulose to ethanol. The pretreatment can lead to changes in the structure and chemical composition of biomass and thus create new features for the pretreated biomass [3]. Liquid hot water (LHW) which is a hydrothermal pretreatment makes the pretreated biomass mainly composed of cellulose and lignin [4]. The residual lignin in the pretreated lignocellulose can negatively influence the enzymatic hydrolysis of cellulose via physical barrier and enzyme adsorption [4–6], and its chemical and structural characteristics are changed due to depolymerization and condensation reactions during LHW pretreatment [7]. Ko et al. [7] extracted lignin from LHW-pretreated hardwoods at four different severities through extensive enzymatic hydrolysis and found that lignin that suffered at more severity of LHW pretreatment exhibited more significant inhibition on enzymatic hydrolysis. The pretreatment type, biomass source, and isolation method determined the characteristics of lignin which would exhibit various impacts on enzymatic hydrolysis [8].

The authors found that a part of cellulose always existed in lignin extractive through the extensive enzymatic hydrolysis of LHW-treated lignocellulose. The residual cellulose in lignin extractive may influence the following research such as enzyme adsorption and enzymatic hydrolysis. In general, the milled wood lignin (MWL) and cellulolytic enzyme lignin (CEL) have been thought to nearly resemble the native lignin. However, the ball-milling process for isolating MWL could result in *β*-ether cleavage [9], and the isolation procedure for CEL could increase phenolic *β*-O-4 content [10] and reduce molecular weight [11]. Recently, She et al. [12] reported that alkali could not change the main structural feature of lignin preparations isolated with 50% dioxane. Although the MWL and CEL have been used as representative sources for structural and chemical analysis of lignin, the application of alkali lignin for further analysis might provide compensatory research. Alkali lignin has seldom been used for characteristic analysis of lignin mainly due to its high amount of nonlignin components, especially hemicellulose [13]. In this study, the relative pure alkali lignin was used to investigate the effect of LHW pretreatment on structural and chemical changes of lignin. The effect of LHW-treated lignin on enzymatic hydrolysis of pure cellulose was investigated. This study was a preliminary try to explore the structural and chemical changes of lignin in the LHW process and would provide useful information for the future research.

## 2. Materials and Methods

### 2.1. Materials

The alkali lignin with 5% moisture (Product ID: 370595) was purchased from Sigma-Aldrich (Shanghai) Trading Co., Ltd. It was composed of 85.0% lignin, 1.3% glucan, and 1.3% xylan. Whatman number 1 filter papers were purchased from Guangzhou Maolin Instrument Co., Ltd. (China). Cellulase whose filter paper activity was 151.7 FPU/g powder was purchased from Imperial Jade Bio-technology Co. Ltd. (China).

### 2.2. Pretreatment

The alkali lignin was pretreated by liquid hot water under the condition of 180°C, 4 MPa, 20 min, which was the optimized operation in the previous study [14]. The ratio of lignin to water was 1 : 20 (g : mL). After pretreatment, the solid residue was air-dried at room temperature and then stored in the desiccator. The LHW-treated lignin was composed of 90.7% lignin and 1.4% glucan.

Whatman number 1 filter papers were cut into pieces in size of less than 1 cm × 1 cm. The pieces were ground at 25000 rpm for 1 min with the swing pulverizer (Guangzhou Daxiang Electronic Machinery Co., Ltd., China) and then stored in the desiccator.

### 2.3. Enzymatic Hydrolysis

The filter paper and lignin mixed in the mass ratio of 0.1 : 0.03 were loaded into a 5 mL Eppendorf tube containing 2.6 mL 0.05 M citrate buffer (pH 4.8). The tubes were sealed with parafilm and fixed on a salver and then placed into a rotatory shaker which was set at 50°C, 150 rpm. The hydrolysis was performed for 72 h with the cellulase loadings of 5, 10, 20, and 40 FPU/g filter paper. The filter papers hydrolyzed under the same conditions without lignin addition were used as the controls. Meanwhile, the filter paper mixing with lignin in the mass ratio of 0.1 : 0.05 was also hydrolyzed with 15 FPU/g filter paper cellulase loadings under the same condition.

### 2.4. Enzyme Activity Assay

The enzyme solution was prepared with 0.198 g cellulase and 10 mL 0.05 M citrate buffer (pH 4.8). The Eppendorf tubes containing 3 mL enzyme solution with and without 30 mg lignin were sealed with parafilm and taped on a salver and then placed into a rotatory shaker at the speed of 150 rpm. After incubating at 50°C for 60 min, the filter paper and CMCase activities of the samples were assayed according to the described methods [15]. The *β*-glucosidase activity was assayed with p-nitrophenyl-*β*-D-glucopyranoside (pNPG) method described in [16].

### 2.5. Adsorption

The filter paper, lignin, and their mixture in the mass ratio of 0.6 : 0.3 were loaded into 50 mL conical flasks containing 20 mL enzyme solution, respectively. The cellulase loading was 20 FPU/g filter paper. The flasks were placed into a rotatory shaker under the condition of 50°C, 150 rpm. The solutions with volume of 200 *μ*L were taken at 5, 10, 15, 20, 40, 60, 80, 100, 120, and 180 min. The protein content was quantified according to the previous method [4]. All of the experiments in this study were in duplicate.

### 2.6. Analytic Methods

Compositional analysis was carried out as the procedure described by the National Renewable Energy Laboratory (NREL) [17]. Lignin treated with and without LHW was coated with a thin layer of gold and then observed with a scanning electron microscope (SEM, S-4800, Hitachi) under an accelerating voltage of 2.0 kV. FTIR test was conducted according to the described KBr pellet technique with a TENSOR 27 Fourier transform infrared spectrometer (Bruker Optics, Germany) [18]. The special surface area, pore size, and total pore volume of lignin were analyzed with the automated surface and porosity analyzer (SI-MP-10/PoreMaster 33, Quantachrome Instruments, USA). Sulfur content of lignin was determined with the elemental analyzer (vario EL cube, Germany). The HPLC system (Waters 2698, USA) equipped with a sugar column (SH1011, Shodex) was applied to measure the sugar concentrations at 50°C with 5 mM H_2_SO_4_ used as the mobile phase at the flow rate of 0.5 mL/min.

## 3. Results and Discussion

### 3.1. Surface Structure

**Figure 1:**
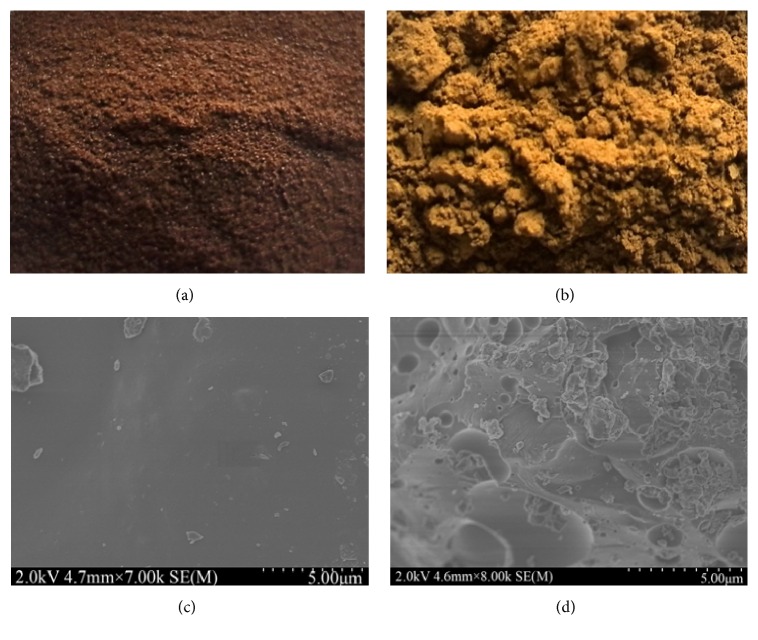
Photographs and SEM observed morphologies of LHW-untreated and LHW-treated lignin, (a) photograph of untreated lignin, (b) photograph of treated lignin, (c) SEM observed untreated lignin, and (d) SEM observed treated lignin.

Liquid hot water is a promising green pretreatment. LHW-treated biomass is mainly composed of cellulose and lignin. LHW pretreatment caused the redistribution of lignin which could influence the enzymatic hydrolysis [19]. In order to discover the changes of lignin during LHW pretreatment, pure alkali lignin was used in this study to avoid the interruption from cellulose and hemicellulose. The pure lignin which was pretreated at 180°C, 4 MPa, for 20 min was air-dried at room temperature to alleviate the influence of high oven temperature on the surface structure [20]. The photographs and SEM observed morphologies of untreated and treated lignin were shown in Figure 1. The untreated lignin was brown and granular (Figure 1(a)), while the treated lignin was yellow and powdered (Figure 1(b)). As for the SEM observed morphologies, the untreated lignin was smooth (Figure 1(c)), while the treated lignin was porous and irregular (Figure 1(d)). These indicated that changes happened to lignin during LHW pretreatment. It was reported that lignin can be melted at temperatures from 170°C to 180°C and redeposited with the temperature decreasing [21].

The surface changes of lignin were quantified with the BET analysis. The BET results of untreated and treated lignin were shown in Table 1. Compared with the untreated lignin, the surface area, total pore volume, and average pore size of treated lignin were raised nearly four times, eight times, and one time, respectively. The increase of surface area meant that the adsorption of cellulase to lignin might be improved. The increase in total pore volume and average pore size might contribute to the access of cellulase to cellulose. It might be deduced that there might be double effects of LHW-treated lignin on the enzymatic hydrolysis. One was that the lignin reduced the cellulolytic efficiency through adsorbing cellulase; the other was that the increase in the pore size of lignin could improve the access of cellulase to cellulose.

### 3.2. FTIR Analysis

**Figure 2:**
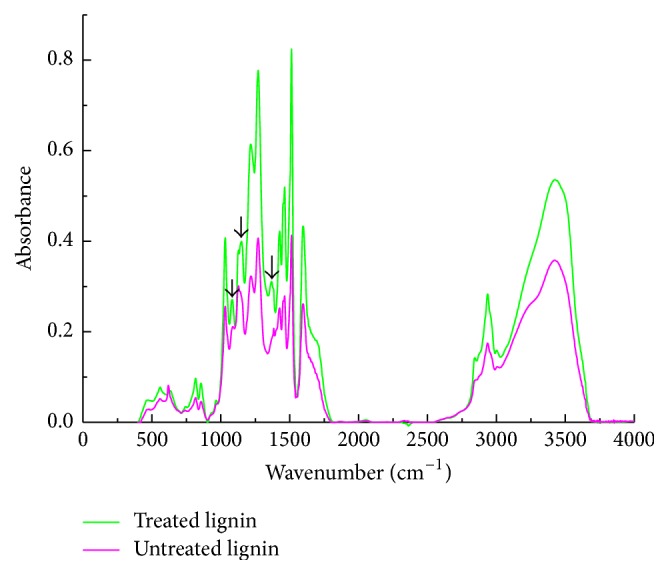
FTIR spectrograms of LHW-untreated and LHW-treated lignin.

The changes of the chemical groups were analyzed with FTIR method. The result was presented in Figure 2. The spectrogram shape of the untreated and treated lignin was almost the same, but the absorbance of treated lignin was stronger than that of untreated lignin, which meant that either new chemical groups were formed or new connections of chemical bonds were built in the LHW process. The band at 3600–3050 cm^−1^ is assigned to O-H stretching vibration which is attributed to the aliphatic and phenolic hydroxyl groups in lignin [22]. The intermolecular and intramolecular hydrogen bonds existed in the aliphatic and phenolic hydroxyl groups, such as O(2)H⋯O(6) intramolecular H-bond, O(3)H⋯O(5) intramolecular H-bond, and O(6)H⋯O(3′) intermolecular H-bond. The stronger absorbance of LHW-treated lignin indicated that, after LHW pretreatment, the H-bond connections of the aliphatic and phenolic hydroxyl groups might be improved or new free hydroxyl groups might be formed in the LHW process. It could be seen from Figure 2 that, after LHW pretreatment, the peaks of lignin at the bands of 1082, 1151, and 1367 cm^−1^ which were marked with the arrows appeared. The bands at 1082, 1151, and 1367 cm^−1^ are assigned to C-O deformation in the secondary alcohols and aliphatic ethers, C-O stretching vibration in the tertiary alcohols, and C-H stretching vibration in the aliphatic methyl and phenol OH, respectively. The assignments of other characteristic peaks were summarized in Table 2. According to the enhanced absorbance of LHW-treated lignin, it could be inferred that some connections of chemical bonds in lignin were broken down, and some new free chemical groups were generated in the process of LHW pretreatment.

### 3.3. The Effect of Lignin on the Enzyme Activity and Adsorption

**Figure 3:**
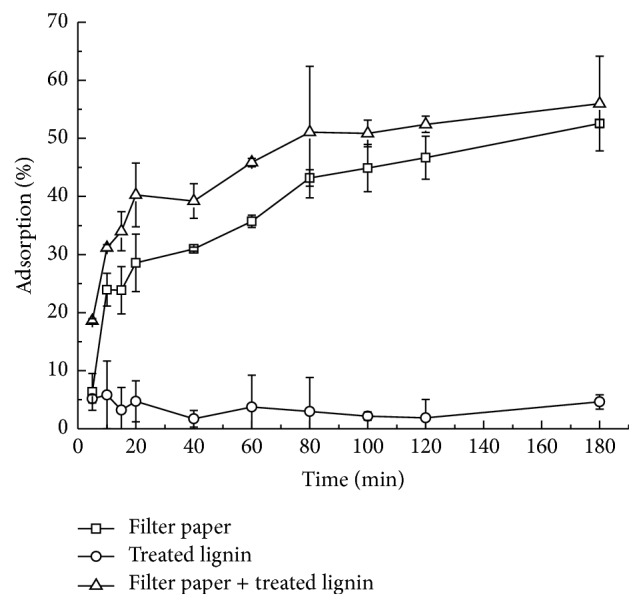
Adsorption of cellulase to LHW-treated lignin, filter paper, and their mixture.

**Figure 4:**
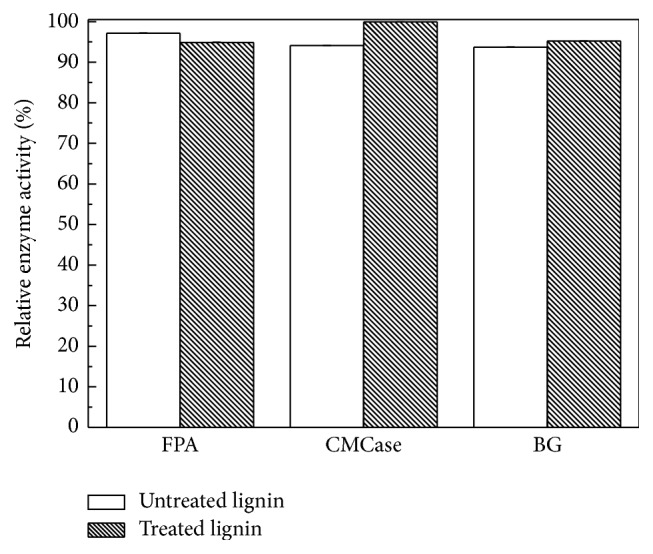
The effect of LHW-untreated and LHW-treated lignin on enzyme activities.

The main inhibition of lignin on enzymatic hydrolysis was thought to be the nonproductive adsorption of cellulase.  Figure 3 showed the adsorption of cellulase to cellulose and treated lignin. The mixture of untreated lignin and citrate buffer presented brown color which interrupted the testing result. Therefore, the adsorption of cellulase to untreated lignin was not shown here. As time goes on, the adsorption of cellulase to treated lignin was at around 5%, while the adsorption of cellulase to filter paper increased from 6.3% to 52.6%. The adsorption of cellulase to the mixture of filter paper and treated lignin was nearly equal to the summation of individual adsorption from filter paper and treated lignin. It meant that the adsorption of cellulase was mainly attributed to the cellulose, which was corresponding to the previous research [23]. Zheng et al. [23] also found that the lignin imposed little impact on the apparent enzyme activity. The same phenomenon was observed in this study and presented in Figure 4. The enzyme activities with and without addition of lignin were measured. The relative enzyme activities were calculated as the ratio of enzyme activities with lignin to that without lignin. After adding untreated and treated lignin, the relative enzyme activities retained more than 90%. The treated lignin hardly put impact on the CMCase activity. The little impact on the enzyme activities might be caused by the little adsorption of cellulase to lignin.

### 3.4. Enzymatic Hydrolysis

**Figure 5:**
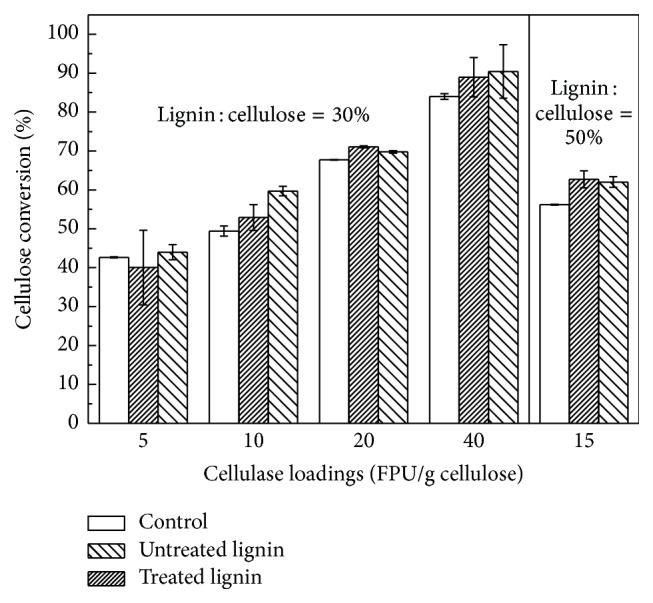
Enzymatic hydrolysis of filter paper in the presence of LHW-untreated and LHW-treated lignin at 50°C for 72 h.

The effect of untreated and treated lignin on the enzymatic hydrolysis of cellulose was carried out in the 5 mL Eppendorf tubes which were sealed with parafilms to avoid the water evaporation. The mass ratio of lignin to cellulose was from 30% to 50% which was in analogy to the contents of lignin and cellulose in the LHW-treated biomass [18]. The ratio of solid to liquid was 5% to relieve the negative effect of viscosity on the enzymatic hydrolysis. The effects of lignin and cellulase loadings on the enzymatic hydrolysis were explored, and the 72-hour enzymatic hydrolyzed results were shown in Figure 5.

Both untreated and treated lignin could improve the enzymatic hydrolysis of cellulose at the lignin loadings of 30% and 50% and cellulase loadings of 5, 10, 20, and 40 FPU/g cellulose. These were contradictory with the previous researches which reported that lignin could inhibit the enzymatic hydrolysis of cellulose [24, 25]. Recently, several researches had reported that lignosulfonate could reduce the nonproductive adsorption of cellulase to lignin through the electrostatic repulsion or acting as the surfactant to enhance the enzymatic hydrolysis of lignocellulose [26–28]. Considering the isolation procedure of alkali lignin, the sulfur content of the alkali lignin used in this study was detected. It showed that the untreated and treated lignin had 1.83% and 1.23% sulfur content, respectively, which were lower than the least sulfur content of the lignosulfonate (5.27%) used in Zhou's research [26]. The improvement of lignin on the enzymatic hydrolysis of cellulose might originate from the small part of lignosulfonate existing in the lignin. Although the treated lignin adsorbed a little cellulase and thus slightly reduced the enzyme activities (Figures 3 and 4), the enzymatic hydrolysis of cellulose was improved (Figure 5). This meant that the negative effect of LHW-treated lignin on the enzymatic hydrolysis could be neglected.

From Figure 5, it also could be seen that the cellulose conversion was increased with the cellulase loading increasing. Whatman number 1 filter paper was used as the pure cellulose to assay the enzyme activity of cellulase in Ghose's method [14]. When the cellulase loading achieved 40 FPU/g cellulose, the conversion of filter paper (the control in Figure 5) did not reach 100% but was just close to 85%. It suggested that the lignin was not the main factor influencing the enzymatic hydrolysis. Although several researches had reported that the removal of lignin could enhance the enzymatic hydrolysis of lignocellulose [29–31], the methods used to remove lignin in their research could induce the structural changes of the lignocellulose. It would be not accurate to only discuss the effect of lignin removal on the enzymatic hydrolysis of lignocellulose. The crystallinity, particle size, nature of the residual lignin and substrate loading, and cellulose accessibility were reported to affect the enzymatic hydrolysis of cellulose [32–36]. The method to extract lignin from lignocellulose could more or less change the native structural characteristics of lignin. The investigation on the impact of lignin extractive on the enzymatic hydrolysis of cellulose may never reflect the real influence mechanism of lignin in the lignocellulose, but it can push the way to discover the truth. A method of reconstructing individual lignin, cellulose, and hemicellulose into a complex should be developed to simulate the lignocellulose. Through the reconstruction, the influence mechanism of lignocellulosic components and structure on enzymatic hydrolysis should be finally disclosed.

## 4. Conclusion

The research on the untreated and treated alkali lignin suggested that the chemical and structural characteristics of lignin were changed in the LHW process. The residual lignin in the LHW-treated lignocellulose which had similar structural and chemical features with the LHW-treated alkali lignin might put its impact on enzymatic hydrolysis of cellulose not via the nonproductive adsorption of cellulase.

## Figures and Tables

**Table 1:** BET measurements of the LHW-untreated and LHW-treated lignin.

	Surface area (m^2^/g)	Total pore volume (cc/g)	Average pore diameter (nm)
Untreated lignin	27.12	0.06	8.78
Treated lignin	125.81	0.53	16.87

**Table 2:** Assignments of characteristic peaks in FTIR spectrogram.

Wavenumber (cm^−1^)	Assignments of characteristic peaks
2937	C-H stretching vibrations in methyl and methylene
1597	Stretching vibrations of aromatic skeleton and C=O
1514	Vibrations of aromatic skeleton
1463	C-H deformations in methyl
1427	Plane deformation of C-H in aromatic skeleton
1271	Stretching vibrations of guaiacyl ring and C=O
1217	Stretching vibrations of C-C, C-O, and C=O
1032	Plane deformations of aromatic C-H, C-O deformations in primary alcohols, and C=O stretching vibrations
856	C-H vibrations in the positions of 2, 5, and 6 out of the plane of guaiacyl units
